# Covariates of Identified Stress and Depression among Seasonal Farmworkers

**DOI:** 10.3390/ijerph14070711

**Published:** 2017-06-30

**Authors:** Beth H. Chaney, Essie Torres

**Affiliations:** Department of Health Education & Promotion, 3105 Belk Building, East Carolina University, Greenville, NC 27858, USA; torrese@ecu.edu

**Keywords:** Latino, seasonal farmworkers, stress, depression

## Abstract

Many noted difficulties of farmworker life result in increased risk for stress and depression. To date, limited research has focused primarily on seasonal farmworkers; much of the prior research examines migrant farmworkers or both groups collectively. This study aims to: (1) describe levels of stress and depression among a sample of *seasonal farmworkers*; and (2) identify if covariates (age, gender, marital status, education level, years of residency, problems obtaining healthcare due to documentation, language barriers, transportation, costs, medical insurance, and stress level) are significant predictors of depressive symptoms. Survey data were collected from 150 Latino seasonal farmworkers. A hierarchical binary logistic regression was conducted to identify significant covariates. The results indicated that the only statistically significant covariates were health insurance coverage (*p* = 0.025) and stress (*p* = 0.008). Those farmworkers without health insurance were 1.8 times more likely than those with health insurance to possess depressive symptoms, while those demonstrating higher stress levels were over 7 times more likely to demonstrate symptoms of depression. The implications of the results are discussed in the manuscript.

## 1. Introduction

Migrant and seasonal farmworkers provide much of the hand labor necessary to support the multibillion dollar agricultural industry within the United States (US) [1,2]. Approximately 2–4 million migrant and seasonal farmworkers are employed on ranches and farms in the US [3], and a majority of these workers are male, Spanish-speaking, and Latino [4,5]. The hired personnel contribute to less than 1% of the wages of all US salary and wage workers, but are essential to the success of US agriculture. As such, migrant and seasonal farmworkers continue to be an economically disadvantaged group in the US [4]. In North Carolina, agriculture ranks among the state’s most vital industries, producing over US $2.2 billion, and at the heart of this industry are the state’s migrant and seasonal farmworkers [6]. A seasonal farmworker is defined as a person whose principal employment is in agriculture on a seasonal basis, and a migrant farmworker meets the same definition, but establishes a temporary home during the period of employment and travels with the season of the crops to do farm work (U.S. Code, Title 42, 1962) [7]. The seasonal farmworker does not move/travel with the crops. The non-agricultural nature of the part-time employment of seasonal farmworkers excludes them from being considered “migrant” workers by the federal definition, and oftentimes results in their ineligibility for federal benefits provided, such as migrant health services [8].

The agricultural industry serves as one of the most dangerous workplace settings in the US [1,8,9,10]. The hazardous work conditions elevate farmworkers into categories documented for poor occupational health and a high incidence of workplace injuries, illnesses, and fatalities [1,5,11,12,13]. Couple this with the many noted difficulties of farmworker life (i.e., living below poverty, unsanitary living and work environments, physical isolation from family and mainstream life, limited access to healthcare), and the result places farmworkers at increased risk for moderate to high levels of stress and depression [1,9,10,13,14]. Moreover, the documented psychological stressors, such as separation from family, discrimination, anxiety around job security and food insecurity, long work hours and lack of essential healthcare, along with the obvious physical stressors of farm labor enhance risk for mental illness [1,10,15,16,17,18]. Previous research indicates that as many as one in four migrant and seasonal farmworkers has experienced a psychiatric disorder episode, which is nearly twice the prevalence of these types of episodes in the general US adult population [18]. Additional research cites 30–40% of migrant and seasonal farmworkers experience clinically significant symptoms of depression [19,20]. Collectively, farmworkers have elevated rates of anxiety, depression, alcohol misuse, and overall poor mental health, which has impacted approximately 20–50% of migrant farmworkers [11,21,22,23]. Furthermore, perceptions of stressors among the farmworker population can be significantly different than perceptions of their healthcare providers [24,25], which ultimately leads to “exacerbating cycles of illness and isolation” [13] (p. 271).

Although some federal benefits may not be provided to the seasonal farmworkers, many do in fact attend farmworker health screenings and programs; however, the inclusion of mental health services in these programs is rare [26]. So rare, that Grzywacz’s [26] conceptual framework for understanding the poor mental health conditions among farmworkers implies that unequal access and unfair treatment is nothing less than social injustice. The specific distinctions between migrant and seasonal farmworkers are often overlooked in published research investigating farmworker living and working conditions, as well as health care access and perceptions of stressors, and given that seasonal farmworkers have elevated stability of residence, linked to subsequent enhanced social support [13], the key differences between the two groups could be relevant when assessing and addressing the health indicators of the population. Examining the associations between identified stressors, such as marital status, legal status, and healthcare access, among the distinct groups of farmworkers is important, since this particular population is underserved, with elevated levels of stress and mental health issues, as compared with the general population. According to Kim-Godwin and Bechtel [13], the detection of various stress levels and depressive symptoms among the farmworker populations is essential for the effective coordination of healthcare services, which links directly to reducing the disparities surrounding access to healthcare for this particular, vulnerable population.

To that end, the primary purpose of this brief report is to: (1) describe the levels of stress and depressive symptoms and associations between the two variables among a sample of *seasonal farmworkers* living and working in Eastern North Carolina (ENC); and (2) identify if covariates (age, gender, marital status, education level, years of residency, problems obtaining healthcare due to documentation, language barriers, transportation, costs, medical insurance, and stress level) are significant factors associated with depressive symptoms among the *seasonal farmworkers* residing in ENC. To date, limited research has focused primarily on seasonal farmworkers, and none, to the authors’ knowledge, has focused only on seasonal farmworkers and stress and depression. Therefore, this analysis provides insight into stress and depression among this distinct group of farmworkers.

## 2. Materials and Methods

*Data Collection.* The data for this analysis were collected via a cross-sectional survey study assessing mental health issues among Latino seasonal farmworkers in ENC. The research team consisted of two university researchers and four bilingual, community health workers, Promotores de Salud, who were trained to effectively recruit, consent, enroll, and collect data from the participating Latino seasonal farmworkers. In addition, the research team collaborated with Greene County Health Care, Inc. (GCHC), which is a federally qualified health center, located in Snow Hill, NC. GCHC has been providing primary care to residents of Greene, Pitt, and Pamlico counties in ENC for over 30 years. The current healthcare delivery system involves three outreach provider teams that conduct regular site visits for hundreds of farmworkers, working in seven surrounding counties. Approximately 99% of patients at GCHC are at or below 200% of the federal poverty line, and 85% are currently uninsured. Furthermore, an estimated 60% of the 30,000+ patients that GCHC serves are classified migrant and seasonal farmworkers [27]. The four community workers, trained to collect the data, also work as outreach providers for GCHC. All of the research protocols were approved by the University and Medical Center Institutional Review Board (IRB) (The IRB code for the approved study is UMCIRB 14-000407 (Continuing Review # -CR00005708).

The convenience sample of participants was recruited from the three-county area, in which GCHC serves, in ENC, and this location has some of the highest farmworker populations in the state [28]. Recruitment involved the community workers/gatekeepers using word-of-mouth and snowball sampling at churches, community mercados (Latino-owned convenience stores), farmworker camps, trailer parks, and soccer teams. Participants were required to be 18 years or older. Recruitment occurred during the spring farming season, and the enrolled participants identified themselves as working farmworkers during this time. Once interested seasonal farmworkers were enrolled, the community workers collected survey data via one-on-one interviews, using the survey instrument as a script. The survey was created in English, translated in Spanish, and field-tested with the Promotores de Salud. Survey data were collected in Spanish using the interview methodology to account for low literacy levels among the farmworker sample. Moreover, each participant was read the consent forms for verbal and written consent, prior to the survey/interview data being collected. Data were collected at farmworkers’ residences or farmworker camps. Lastly, each participant received a $10 Wal-Mart gift card for participating in the survey data collection.

*Sample and Setting.* All of the participants lived in Greene or Pitt Counties, located in Eastern North Carolina. North Carolina has one of the fastest growing Latino populations in the country, and is estimated to rank as the sixth most populous farmworker state in the US [28]. According to research conducted by Kim-Godwin and colleagues [13], most of these farmworkers are located in the Eastern and military portions of the state. Eastern North Carolina is home to residents who have the poorest health statistics in North Carolina, and this is referring to the general population [29]. The seasonal farmworkers recruited for the current analysis indicated having a permanent address/residence in the targeted research area. The study did not specifically target male farmworkers; therefore, both men and women seasonal workers, 18 years and older, were eligible to participate in the study. Survey data were collected by the trained Spanish-speaking community workers during April–May 2014 from 150 Latino seasonal farmworkers.

*Measures.* To measure participant levels of stress, a shortened version (8-items) of an instrument developed to assess stress among migrant and seasonal farmworkers was used [9]. This scale includes Spanish validated items assessing stress associated with occupational health, with previous demonstrations of effective utility and validation in the farmworker population [9]. It was developed, and has published results on the validity of the measure [9], with a population of Latino migrant and seasonal farmworkers in Eastern North Carolina. Collectively, the items measure stress related to work availability, unfair treatment at work, lack of money, medical bills, health/sickness, documentation, and job and/or family problems. The item response options were dichotomized to “yes” or “no” responses, and sum scores of “yes” responses were calculated on a scale from 0–8 for each farmworker. The Kuder–Richardson 20 (KR-20) reliability coefficient for the dichotomous data produced from using these 8-items was 0.731, which suggests moderate and acceptable levels of reliability [30]. Higher sum scores reflect higher levels of stress, and scores were categorized to “more than 6” and “less than 6” to reflect levels of stress among the sample. The cutoff score was determined by estimating the area under the received operating characteristic (ROC) curves, with the screening measure being the standard sumscore of the stress measure and the outcome measure being the recoded dichotomous variable of “more than 6” or “less than 6” to assess the area under the curve (AUC) for screening measure validity [31]. The AUC test result for the ROC curve for the proposed cutoff was 0.992 (*p* < 0.001; CI 0.906–1.00), which suggests that the cutoff appropriately identifies those with higher levels of stress, from those with lower levels of stress [31].

*Depressive symptoms* among Latino seasonal farmworkers were measured with a 10-item shortened version of the Chaney et al. [9] depression scale, created from the Spanish-validated Center for Epidemiologic Studies Depression Scale (CES-D), among others. The scale was developed with migrant and seasonal farmworkers in Eastern NC, and the comprehensive process for adapting previously-used items, such as the CES-D, specifically for these farmworkers is published elsewhere [9]. Additionally, the KR-20 reliability coefficient for the 10-items was calculated as 0.831. The items were dichotomized to “yes” or “no” responses, and measured “feelings of sadness, hopelessness, and depression” associated with family, work and/or health problems, and living and/or working conditions. In addition, items assessed sleep loss due to feelings of sadness, depression, and hopelessness, and any difficulties carrying out normal work- and home-life activities. The “yes” responses were summed among each case to create a sum score, and these were categorized to “more than 8” and “less than 8” to reflect levels of depressive symptoms among Latino seasonal farmworkers. Higher scores reflect more symptoms of depression. To determine the categories for levels of depressive symptoms, the area under the received operating characteristics (ROC) curve was calculated. The screening measure was the standard sumscore measure, while the outcome measure was the recoded dichotomous variable of “more than 8” or “less than 8” to assess the area under the curve (AUC) for screening measure validity [31]. The AUC test result for the proposed cutoff was 0.892 (*p* < 0.001; CI 0.806–0.902), which suggests the cutoff appropriately identifies those with higher levels of depressive symptoms, from those with lower levels of depressive symptoms [31].

*Participant characteristics (covariates)* were assessed, and included age, categorized as 18–26 years, 27–34 years, 35–42 years, 43–50 years, 51–60 years, and 61 years and older. Marital status response options included “single”, “free-union”, “married”, “divorced”, “separated”, and “widowed”. Completed education level was categorized as “no formal education”, “Elementary School”, “Junior High School”, “High School/GED”, “College (2 year/4 year)”, and “Graduate or Professional”. Income was measured by assessing average monthly income for each respondent. Health insurance response options were a dichotomy of “yes” or “no”, as were the response options for items assessing problems obtaining health care due to lack of time, language barriers, lack of insurance, lack of transportation, money issues, documentation issues, and child care issues. Years of residency were categorized into “less than 1 year”, “1–3 years”, “4–10 years”, “11–20 years”, and “21+ years”. Age, marital status, education level, and years of residency were categorized as personal covariates (i.e., demographic characteristics) of the participants that were selected for further analysis in relation to participant stress and depressive symptoms. Additionally, health insurance access and the various problems obtaining care that were assessed as covariates have been linked to elevated levels of stress and depressive symptoms in the farmworker population, and were therefore further analyzed in this study.

*Data Analysis.* Data were analyzed using the Statistical Package for Social Sciences (SPSS) v24 (IBM Corp, Released 2016. IBM SPSS Statistics, Armonk, NY, USA), and descriptive statistics were calculated to assess participant characteristics. Correlational analyses were conducted to evaluate the relationship between levels of stress and depressive symptoms, and cross-tabulations, using chi-square analyses, were performed to assess the varying distributions of targeted variables. In order to evaluate the ability of the identified covariates to predict depressive symptoms among the farmworker sample, a hierarchical binary logistic regression was conducted. Block 1 assessed personal covariates: age, marital status, education level, and years of residency, while block 2 assessed health-related stressors as covariates: health insurance, problems due to lack of time, language barriers, lack of insurance, lack of transportation, money issues, documentation issues, child care issues, and levels of stress.

## 3. Results

### 3.1. Participants

Data were collected from a sample of 150 Latino seasonal farmworkers. Respondents ranged in age from 18 to 76 years old (mean age = 39, SD = 12.669), and a plurality of farmworkers were between the ages of 35–42 years old (n = 40, 26.6%). Most of the farmworkers were from Mexico (73%), spoke Spanish as their primary language (80%), and identified as female (55.3%). A majority of the sample had lived in Greene or Pitt Counties for at least 11–20 years (40%), with approximately 35% indicating permanent residency for 4–10 years. Additionally, many were married (43.3%), with either elementary school (33.3%) or junior high school (32.7%) education levels. A large majority had no health insurance (78%), but only 30.2% indicated having problems receiving health care. For those indicating having problems obtaining health care, 22.1% cited lack of money as a reason, while 16.8% cited the lack of insurance. Current occupation/job was assessed, and most were employed at that time as a farmworker (34%), but others were employed in non-agricultural jobs during this time; including: construction (13.3%), housekeeping (8.7%), and retail (6.7%). Average monthly income ranged from less than U.S. $500 to more than U.S. $1000; a majority fell into the U.S. $500–$700/month range. Refer to Table 1 for detailed information on the demographics of the seasonal farmworker sample.

### 3.2. Data Analysis Results

A Pearson product-moment correlation between levels of stress and depression sum scores indicated a positive association (*r* = 0. i.e., 668, *p* < 0.001); as stress levels increased, so did documented depressive symptoms. Only 12.7% of the sample had a stress sum score of “6 or more”, and out of that percentage, a majority were female (74%), with 4–10 years of residency (47.3%), married (31.6%), having no health insurance (100%), within the $500–$700 monthly income range (47.3%), and educated at the elementary school level (47.3%). Additionally, 11.3% of the sample indicated having depression (sum scores of “8 or more”), with most being female (64.7%), residents between 11 and 20 years (52.9%), free union (47%), educated at the elementary school level (53%), having no insurance coverage (100%), and in the monthly income ranges of less than U.S. $500 (29.4%) and between U.S. $500–$700 (29.4%). Table 2 provides the results from the bivariate chi-square analyses of the selected variables. The results indicated that only health insurance access (χ^2^ = 5.90, df = 2, *p* = 0.05) and lack of documentation (χ^2^ = 9.15, df = 2, *p* = 0.01) resulted in statistically significant differences between participants identified as having high stress levels versus those with lower stress levels.

The hierarchical regression analysis indicated the predictive model was statistically significant (χ^2^ = 32.905, df = 18, *p* = 0.017), and explained between 19.8% (Cox and Snell R-square) and 38.9% (Nagelkerke R-square) of variance for classifying those who exhibited depressive symptoms. The model also correctly identified 73.1% of all cases. The only statistically significant covariates that contributed to the proposed model were health insurance coverage (*p* = 0.025) and stress (*p* = 0.008). Those farmworkers without health insurance were 1.8 times more likely than those with health insurance to possess depressive symptoms (Exp(B) = 1.834), while those demonstrating higher stress levels were over 7 times more likely to demonstrate symptoms of depression ((Exp(B) = 7.321). See Table 3 for regression model results.

## 4. Discussion

Most of the research conducted within the farmworker population has aggregated data from the two distinct groups of farmworkers: migrant and seasonal. Given the differences between migrant and seasonal farmworkers [7,13], describing the mental health issues of each distinct group is warranted. Therefore, the primary aim of this research was to describe stress and depressive symptoms among 150 Latino *seasonal farmworkers* living in Eastern North Carolina. In addition, the study sought to identify significant covariates of depressive symptoms within the sample of seasonal farmworkers.

Approximately 13% of the sample documented high levels of stress, while 11.3% identified higher levels of depressive symptoms. In comparison to prior research on migrant and seasonal farmworker stress levels [9,32], these levels are relatively low. However, compared to the general U.S.-born Latino population, Latino immigrants tend to report lower levels of stress and anxiety, which is sometimes called the “immigrant paradox”, capturing the idea that foreign nativity can serve as a protective factor against mental illness or other psychiatric disorders [33]. Additionally, compared to the prevalence rates of depressive symptoms of the general population (ranging by state and territory, 5.3–13.7%) and the depression diagnosis rates (15.7%), this sample of seasonal farmworkers demonstrated similar levels of depressive symptoms [34]. Seasonal farmworker lifestyles allow for limited mobility and permanent residence (compared to migrant workers), less frequent strenuous farm labor (given the ability to secure non-agricultural jobs in harvest off-seasons), and more social support, as seasonal farmworkers become a part of a community [13]. These varying aspects of migrant and seasonal farmworkers may very well contribute to the lower levels of stress and depression documented in this study.

Moreover, it is important to note that the current sample of seasonal farmworkers is mostly made-up of workers immigrating from Mexico and more women than men. Cultural differences among multiple ethnic groups have been found to impact overall perceived stress and mental health [35]. Specifically, previous research has suggested that stress impacts the mental and physical health of various ethnic/cultural groups differently, due to the ways that social support within those groups is configured [35]. Therefore, the impact of stress on one’s mental health status may very well be contingent on cultural identity, and in a study conducted by Shavitt and colleagues (2016), Mexican Americans were more likely to seek health advice from friends and family, and were found to have more social support to help buffer against stress, than other ethnic groups. Given that most farmworkers, nationwide, are males [1], and this sample is predominantly female, this study provides unique insight into the levels of stress, depressive symptoms, and factors contributing to both, from the perspective of female seasonal farmworkers. As such, a majority of the farmworkers indicating elevated levels of stress and depression were female, living within ENC, and with no health insurance coverage.

Although the results of the analysis indicate a low prevalence of stress and depression among the seasonal farmworkers, the results of stress levels being highly correlated with depressive symptoms are consistent with prior research [14]. Specifically, as the documented stress increased, so did the number of depressive symptoms among the seasonal farmworker sample. The harsh reality of this farmworker population involves this group being economically disadvantaged, with limited access to health insurance, low education levels, and overall substandard living conditions, due to financial issues among the group. Although the seasonal farmworkers are more integrated into society through permanent community residence, these members of society are continually plagued by low wages, language barriers, inadequate health care, and the physical and psychological stressors of being a farmworker [1,14]. Furthermore, seasonal farmworkers fall into the gap of not being covered by federal health services, as exampled by the exemptions of the Patient Protection and Affordable Care Act (ACA). Seasonal farmworkers, working less than 120 days a year on farms and ranches, do not have to be covered by “small” agricultural employers, and typically cannot afford the state health insurance exchanges, although migrant farmworkers through H2-A programs should technically be eligible for coverage under the ACA [36].

The regression model results indicated that health insurance access and levels of stress were significant predictors of depressive symptoms in the posed model. As noted above, the relationship between stress and depression has long been examined within the farmworker population [14], and the positive relationship has been established between these two variables. However, the only other significant covariate within the model was health insurance access. Those without health insurance were 1.8 times more likely to demonstrate higher levels of depressive symptoms than those with health insurance. Arcury and Quandt’s [1] extensive review of health care access and health care utilization patterns among migrant and seasonal farmworkers supports this specific finding in the current study. Health care access, including insurance coverage, is a fundamental source of stress in this underserved, vulnerable population. This finding held true when migrant farmworkers were taken out of the analysis, and therefore reflects the current situation of seasonal farmworkers living in ENC. Probably most interesting about this study’s findings is the fact that many covariates, such as marital status, education level, years of residency, problems obtaining healthcare due to discrimination, documentation, language barriers, and transportation, were not significant predictors of depressive symptoms among the seasonal farmworker sample. As noted in the introduction, many studies which have assessed both migrant and seasonal farmworker groups collectively, have found that many of these covariates predict farmworker stress and depression. Given that this study did not support those findings, more research on stress and depression between the distinct groups of farmworkers is warranted.

*Limitations*. The study methodology used to compile the small convenience sample of Latino seasonal farmworkers is subject to selection bias. Therefore, a generalization of the findings to all seasonal farmworkers is limited, simply due to sample composition. Self-reported data interjects issues of reliability and validity; however, the methods used to collect these data (i.e., interview-assisted surveys) sought to improve the validity and reliability of the data secured. Another potential limitation is that the nature of this study (i.e., cross-sectional study) and analysis plan does not lend itself to the establishment of causal relationships. Therefore, more research is needed to identify causal relationships between the identified significant covariates and depression among seasonal farmworkers. Lastly, it is important to note that the measures used in this study were adapted from previously-used instruments, and therefore the direct comparison of results between this study and other studies (not using the exact same measures) cannot be made.

## 5. Conclusions

The results of the study indicate a need for more efforts to address mental health issues in farmworker communities, although addressing these issues can be rather challenging. The current study’s implications support teaching positive coping mechanisms for addressing stressful situations. Health education programs to provide effective strategies for promoting positive coping among farmworkers (i.e., seeking support from family and friends, consulting trained counselors, engaging in healthy behaviors, such as physical activity) could result in better stress management skills. The working conditions associated with elevated stress levels could be improved if current required regulations related to sanitation were enforced. In addition, mental health services are provided to farmworkers at the local federally qualified healthcare center; therefore, this is a valuable resource for the farmworkers in this study. To address the lack of insurance barrier to seeking care, more nontraditional programs, such as Spanish-language mental health hotlines, free mobile units in these communities (funded through local city council or possibly local universities with medical and/or graduate counseling programs with graduate students needing/wanting experience), or late night hours at local community clinics for those farmworkers in need, could be implemented to assist in improving the mental health of farmworkers [10].

Given that healthcare access was the single, significant variable in the current study, strategies for improving access to healthcare for this particular seasonal farmworker population are needed. Specifically, better marketing of the services offered to all seasonal farmworkers at GCHC could help improve the utilization of these services. This Federally Qualified Health Center provides comprehensive primary care in six locations throughout the rural areas of ENC, with a special focus on uninsured, underserved, and underinsured patients. Additionally, communicating these findings to the Director of Outreach Services at the health center provides unique insight into the perceptions of seasonal farmworkers and their (lack of) access to healthcare. Engaging seasonal farmworkers as locals/community members versus migrant farmworkers, whose care needs are temporary (based on crop seasons), can better inform healthcare providers on how to best meet the unique medical needs of the seasonal farmworkers who reside in the surrounding areas.

## Figures and Tables

**Table 1:** Participant characteristics.

Age	n (%)
18–26 y/o	24 (16)
27–34 y/o	34 (22.7)
35–42 y/o	40 (26.6)
43–50 y/o	24 (16)
51–60 y/o	20 (13.4)
61 and older	8 (5.3)
Gender	
Female	83 (55.3)
Male	67 (44.7)
Nationality	
USA (US & Puerto Rico)	8 (5)
South American (Columbia, Uruguay, Venezuela)	13 (9)
Central America (Guatemala, Honduras, Panama, San Salvador)	12 (8)
Mexico	110 (73)
Years of Residency	
Less than 1 year	6 (4)
1–3 years	6 (4)
4–10 years	52 (34.7)
11–20 years	60 (40)
21+ years	25 (16.6)
Marital Status	
Single	38 (25.3)
Free-union	36 (24)
Married	65 (43.3)
Divorced	3 (2)
Separated	2 (1.3)
Widowed	3 (2)
Education	
No formal education	9 (6)
Elementary School	50 (33.3)
Junior High School	49 (32.7)
High School/GED	24 (16)
College (2 year/4 year)	9 (6)
Graduate or Professional	7 (4.7)
Health Insurance Coverage	
Yes	30 (20)
No	117 (78)
Current Job	
Agricultural Work (plant farms, field crop, livestock work, etc.)	51 (34)
Packing/canning (meat, fruit vegetables)	3 (2)
Logging/forestry	2 (1.3)
Hotel, motel, or restaurant	17 (11.3)
Retail (stores, shops)	10 (6.7)
Services (health care, social services)	7 (4.7)
Housekeeping	13 (8.7)
Government Office	2 (1.3)
Construction	20 (13.3)
Education	2 (1.3)
Average Monthly Salary	
Less than $500	23 (15.3)
$500–$700	46 (30.7)
$701–$1000	47 (31.3)
More than $1000	27 (18)
Problems Obtaining Health Care	
Yes	45 (30.2)
No	102 (68.5)
Identified Problems Assessing Health Care (Yes responses)	
Lack of time	13 (8.7)
Language barriers	10 (6.7)
No medical insurance	25 (16.8)
Transportation	9 (6)
Money	33 (22.1)
Lack of documentation	8 (5.4)
Child care	1 (0.07)

y/o: year old.

**Table 2:** Bivariate analyses of covariates between participants with high stress (HS) versus low stress (LS) and high levels of depressive symptoms (HD) versus low levels of depressive symptoms (LD).

Variables	LS	HS	χ^2^ (df)	Sig.	LD	HD	χ^2^ (df)	Sig.
Gender	Male (41.3%)	Male (9.3%)	2.96 (1)	0.085	Male (40.7%)	Male (4%)	0.681 (1)	0.409
	Female (46%)	Female (3.3%)			Female (48%)	Female (7.3%)		
Marital status	Single (22%)	Single (1.3%)	4.35 (2)	0.63	Single (24%)	Single (1.3%)	9.55 (3)	0.145
	Free union (21.3%)	Free union (5.3%)			Free union (19.3%)	Free union (5.3%)		
	Married (40%)	Married (3.3%)			Married (40%)	Married (3.3%)		
	Divorced (1.3%)	Divorced (0.6%)			Divorced (1.3%)	Divorced (0.6%)		
	Separated (1.3%)	Separated (0.6%)			Separated (1.3%)	Separated (0.6%)		
	Widowed (2%)	Widowed (0%)			Widowed (0.6%)	Widowed (0%)		
Education level	None (6.7%)	None (0.6%)	4.53 (3)	0.605	None (6%)	None (1.3%)	6.86 (3)	0.334
	Elementary (27.3%)	Elementary (6%)			Elementary (27.3%)	Elementary (6%)		
	Junior High (29.3%)	Junior High (3.3%)			Junior High (29.3%)	Junior High (3.3%)		
	High School (42.7%)	High School (2.7%)			High School (15.3%)	High School (0.6%)		
	College (6%)	College (0%)			College (6%)	College (0%)		
	Graduate (4.6%)	Graduate (0%)			Graduate (4.7%)	Graduate (0%)		
Years of residency	>1 year (2%)	>1 year (0.6%)	11.3 (4)	0.186	>1 year (2%)	>1 year (0.6%)	6.56 (4)	0.584
	1–3 years (4.7%)	1–3 years (1.3%)			1–3 years (6%)	1–3 years (0%)		
	4–6 years (14.7%)	4–6 years (0.6%)			4–6 years (14%)	4–6 years (1.3%)		
	7–10 years (14%)	7–10 years (5.3%)			7–10 years (17.3%)	7–10 years (2%)		
	11–15 years (21.3%)	11–15 years (1.3%)			11–15 years (18.7%)	11–15 years (4%)		
	16–20 years (15.3%)	16–20 years (2%)			16–20 years (15.3%)	16–20 years (2%)		
	21–30 years (8.7%)	21–30 years (0.6%)			21–30 years (9.3%)	21–30 years (0%)		
	30+ years (6.7%)	30+ years (0.6%)			30+ years (6%)	30+ years (1.3%)		
Health insurance	No (66%)	No (12.7%)	5.90 (2)	0.050 *	No (68.7%)	No (11.3%)	5.19 (2)	0.074
	Yes (21.3%)	Yes (0%)			Yes (20%)	Yes (0%)		
Lack of Time	No (78.7%)	No (12.7%)	2.24 (2)	0.326	No (80.7%)	No (10.7%)	0.89 2(2)	0.64
	Yes (8.7%)	Yes (0%)			Yes (8%)	Yes (0.6%)		
Language barriers	No (82%)	No (10.7%)	2.63 (2)	0.269	No (83.3%)	No (9.3%)	3.53 (2)	0.172
	Yes (5.3%)	Yes (2%)			Yes (5.3%)	Yes (2%)		
Lack of transportation	No (82%)	No (12%)	0.266 (2)	0.875	No (84%)	No (10%)	1.71 (2)	0.425
	Yes (5.3%)	Yes (0.6%)			Yes (4.7%)	Yes (1.3%)		
Money issues	No (67.3%)	No (10%)	0.268 (2)	0.874	No (69.3%)	No (8%)	1.05 (2)	0.59
	Yes (20%)	Yes (2.7%)			Yes (19.3%)	Yes (3.3%)		
Child care issues	No (86.7%)	No (12.7%)	0.392 (2)	0.822	No (88.7%)	No (10.7%)	8.47 (2)	0.14
	Yes (0.6%)	Yes (0%)			Yes (0%)	Yes (0%)		
Document-ation issues	No (84%)	No (9.3%)	9.151 (2)	0.01 **	No (84.7%)	No (9.3%)	5.12 (2)	0.077
	Yes (3.3%)	Yes (2.7%)			Yes (4%)	Yes (2%)		

* *p*-value: statistically significant at the α = 0.05 level; ** *p*-value: statistically significant at the α = 0.01 level.

**Table 3:** Logistic regression model for identifying significant covariates for depressive symptoms among participants.

		Exp (B)	95% CI	df	Sig.
Depression					
Block 1	Age ^1^	0.995	0.935, 1.059	1	0.878
	Marital status ^2^	0.879	0.512, 1.511	1	0.641
	Education level ^3^	1.497	0.633, 3.540	1	0.359
	Years of residency ^4^	0.734	0.477, 1.130	1	0.106
Block 2	Health insurance ^5^	1.834	0.834, 2.458	1	* 0.025
	Lack of Time ^6^	0.726	0.122, 4.321	1	0.725
	Language barriers ^7^	0.500	0.36, 6.857	1	0.604
	Lack of transportation ^8^	0.531	0.119, 2.361	1	0.406
	Money issues ^9^	0.335	0.066, 1.707	1	0.188
	Child care issues ^10^	0.148	0.028, 0.771	1	0.123
	Documentation issues ^11^	0.188	0.335, 0.066	1	0.189
	Stress ^12^	7.321	1.689, 31.729	1	* 0.008

^1^ Age reference category: 35–42 years old; ^2^ Marital status reference category: married respondents; ^3^ Education reference category: no education respondents; ^4^ Years of residency reference category: 11–20 years; ^5^ health insurance reference category: none; ^6–11^ Reference category: yes responses; ^12^ Stress: <6 sum scores; * statistically significant at 0.05 *p*-value.
